# Supplementary material for: Addressing critiques refines global estimates of reforestation potential for climate change mitigation
Source: Nat Commun. 2025 Jun 11;16:4572. doi: 10.1038/s41467-025-59799-8 (PMC12159164; doi:10.1038/s41467-025-59799-8)
Supplement: Supplementary file 1 — Supplementary Information [file 41467_2025_59799_MOESM1_ESM.pdf]

Supplementary Information for:

Addressing critiques refines global estimates of reforestation potential for climate change mitigation

Kurt A. Fesenmyer[1*], Erin E. Poor[2], Drew E. Terasaki Hart[3,4], Joseph W. Veldman[5], Forrest Fleischman[6], Pooja Choksi[6], Sally Archibald[7], Mohammed Armani[8], Matthew E. Fagan[9], Evan C. Fricke[10], César Terrer[10], Natalia Hasler[11], Christopher A. Williams[12], Peter W. Ellis[13], Susan C. Cook-Patton[3*]

[1]Tackle Climate Change, The Nature Conservancy, Boise, ID, USA.
[2]Tackle Climate Change, The Nature Conservancy, Denver, CO, USA.
[3]Tackle Climate Change, The Nature Conservancy, Arlington, VA, USA.
[4]CSIRO Environment, Brisbane, QLD, Australia.
[5]Department of Ecology and Conservation Biology, Texas A&M University, College Station, TX, USA.
[6]University of Minnesota, Department of Forest Resources, St. Paul, MN, USA.
[7]School of Animal, Plant and Environmental Sciences, University of the Witwatersrand, Johannesburg 2050, South Africa.
[8]College of Agriculture and Natural Resources, Kwame Nkrumah University of Science and Technology, Kumasi, Ghana.
[9]Department of Geography and Environmental Systems, University of Maryland, Baltimore County, Baltimore, MD, USA.
[10]Department of Civil and Environmental Engineering, Massachusetts Institute of Technology, Cambridge, MA, USA.
[11]George Perkins Marsh Institute, Clark University, Worcester, MA, USA.
[12]Graduate School of Geography, Clark University, Worcester, MA, USA.
[13]Tackle Climate Change, The Nature Conservancy, Portland, ME, USA.
*Corresponding authors: kurt.fesenmyer@tnc.org, susan.cook-patton@tnc.org

Content list:

Supplementary Table 1: Categories of spatial data recorded for 89 previously published maps from 2011-2022, with example publications by application type

Supplementary Table 2: Method details, area, and mitigation for forest potential, maximum reforestation potential, and constrained reforestation potential in global map products and this analysis

Supplementary Table 3: Exclusion and overlay datasets used in this analysis

Supplementary Table 4: Area of intersection (agreement) for this analysis and other global forest potential and maximum or constrained reforestation potential maps

Supplementary Table 5: Composite secure land tenure overlay data sources and sensitivity analysis results

Supplementary Figure 1: Reforestation map coverage

Supplementary Figure 2: Forest potential map agreement

Supplementary Figure 3: Constrained reforestation potential map agreement

Supplementary Figure 4: Forest potential map

Supplementary Figure 5: Forest loss:gain ratio, 2000-2020

Supplementary Figure 6: Avoiding social conflicts scenarios

Supplementary Figure 7: Ecosystem services scenarios

Supplementary Figure 8: Government policy scenarios

Supplementary Figure 9: Sensitivity testing – forest potential

Supplementary Figure 10: Sensitivity testing - exclusions

Supplementary Figure 11: PRISMA diagram for literature review methods

Supplementary Table 1 | Categories of spatial data recorded for 89 previously published maps from 2011-2022, with example publications by application type. Suitability criteria are used to delineate areas included in the forest potential or maximum/constrained reforestation potential. Exclusions are used to remove or mask areas from the forest potential or maximum/constrained reforestation potential. Overlays are used for further refinement or prioritization of reforestation potential. Individual details on each of the 89 published maps we reviewed is provided via our 'Data Availability' section.

Dataset category	Suitability criteria example	Exclusion example	Overlay example
Existing forest occurrence or condition (local)	Keles et al. 2013 [1]	Erbaugh et al. 2020 [2]	Pascual et al. 2021 [3]
Deforested land or areas of forest loss (local)	Orsi et al. 2011 [4]		Zeng et al. 2020 [5]
Biome, ecoregion, or potential natural vegetation	Graves et al. 2020 [6]		
Contemporary (1980-present) forest pattern or configuration (e.g., patch size, neighborhood statistics)	Crouzeilles et al. 2020 [7]		Crouzeilles et al. 2019 [8]
Forest regrowth (including quasi-spatial deductions)		Fargione et al. 2018 [9]	
Carbon sequestration			Gourevitch et al. 2016 [10]
Another forest or reforestation potential map	Wilkes et al. 2020 [11]		
Tree plantation	Echeverria et al. 2013 [12]	Busch et al. 2019 [13]	West et al. 2020 [14]
Surface water (including distance metrics)		Uribe et al. 2014 [15]	Syartinilia et al. [16]
Urban areas (including distance metrics)	Teo et al. 2021 [17]	Gopalakrishna et al. 2022 [18]	Zheng et al. 2022 [19]
Croplands	Molin et al. 2018 [20]	Nunes et al. 2017 [21]	Santos et al 2020 [22]
Pasture (including non-native grassland vegetation)	Lemos et al. 2021 [23]		Fargione et al. 2018 [9]
Grasslands (native vegetation type)		Cook-Patton et al. 2020 [24]	
Wetlands		Burke et al. 2021 [25]	
Permanent ice or snow		Drever et al. 2021 [26]	
Rock, barren land, or sand (including minelands, arid lands)	Aguirre-Salado et al. 2017 [27]	Strassburg et al. 2020 [28]	
Shrublands		Cook-Patton et al. 2020 [24]	
Terrain (e.g., elevation, slope, aspect)	Gopalakrishna et al. 2022 [18]	Zhang et al. 2020 [29]	Lemos et al. 2021 [23]
Topographic position (e.g., floodplains, riparian areas)	Poccard-Chapuis et al. 2021 [30]		Ribeiro et al. 2020 [31]
Soils	Barnett et al. 2016 [32]	Cook-Patton et al. 2020 [24]	Cook-Patton et al. 2020 [24]

Dataset category	Suitability criteria example	Exclusion example	Overlay example
Land status or tenure (e.g., protected areas or reserves, land ownership)	Pascual et al. 2021 [3]	Drever et al. 2021 [26]	Busch et al. 2019 [13]
Roads (including distance metrics)		Chen et al. 2021 [33]	Uribe et al. 2014 [15]
Biodiversity			Cook-Patton et al. 2020 [24]
Current climate			Brancalion et al. 2019 [34]
Future climate	Bastin et al. 2019 [35]		Bastin et al. 2019 [35]
Albedo effects			Drever et al. 2021 [26]
Wildfire (e.g., burn history)	Graves et al. 2020 [6]	Drever et al. 2021 [26]	
Economic factors (e.g., restoration cost, commodity prices)			Polglase et al. 2013 [36]
Social factors (e.g., demographics)			Erbaugh et al. 2020 [2]
Political factors (e.g., political boundaries, spatially-explicit policy goals)			Greve et al. 2013 [37]
Legal factors (e.g., Brazilian Native Vegetation Protection Law)			Strassburg et al. 2019 [38]
Other factors	Chomphosy et al. 2021 [39] focus on oil and gas well pads	Ribeiro et al. 2020 [31] exclude previous restoration lands and forestry lands	Greve et al. 2013 [37] overlay groundwater recharge areas

Supplementary Table 2 | Method details, area, and mitigation for forest potential, maximum reforestation potential, and constrained reforestation potential in global map products and this analysis. This table provides an overview of the methods for mapping reforestation potential in the available global maps, including how forest potential is mapped and which exclusions are applied to that forest potential map. Total area of forest potential and reforestation potential are provided for each product, where relevant, along with mitigation potential.

Map	Forest Potential		Exclusions						Reforestation Potential				
	**Criteria description**	**Global area (Mha)**	**Existing forest**	**Cropland**	**Pasture**	**Wetland**	**Urban**	**Other**	**Type**	**Global area (Mha)**	**Global mitigation**	**Mitigation unit**	**Mitigation comment**
FLRO	> 45% potential canopy cover (closed forest)	4,253	(1)	(2)	*None*	(3)	(4)	*None*	Constrained	514	-	-	
	> 25% potential canopy cover (closed and open forests)	5,706								-	-	-	
	> 10% potential canopy cover (closed and open forest, woodlands)	7,506	(1)	(2)	*None*	(3)	(4)	*None*		2,509	-	-	
Griscom et al. 2017	*(Uses FLRO > 25% potential canopy cover)*	5,706	(5)	(2)	*None*	(3)	(4)	Boreal forests (for albedo), grassy biomes, background forest regrowth rate 2016-2030	Constrained	678	2,761	TgC/yr	Growth rate from lit. review of AGB + BGB by tropical, temperate climate domain (SOC also considered in tropical domain), with additional deductions of mitigation estimates of grazing NCS pathways (to avoid double-counting)
Bastin	> 10% potential tree cover	8,682	(6)	(7)	*None*	*None*	(7)	*None*	Constrained	1,772	-	-	Bastin et al. only report mitigation for canopy cover restoration (potential minus existing tree cover), not forest restoration
		8,682	(6)	(8)	*None*	None	(7)	*None*		1,657	-	-	
Cook-Patton et al. 2020	*(Uses FLRO > 25% potential canopy cover)*	5,706	*(Uses Griscom et al. 2017)*							678	2,432	TgC/yr	Modeled annual natural forest regrowth rates over 30 years for AGB + BGB + SOC pools
Strassburg	Forest with >15% tree cover in predominant 'original ecosystem' type	6,160	(9)	*None*	*None*	(9)	(9)	Bare ground, ice/snow	Maximum	1,537	-	-	
Zheng et al. 2022	*(Uses FLRO > 25% potential canopy cover)*	5,706	*(Uses Griscom et al. 2017 removes urban and cropland expansion areas (2020-2100) in 2 land-use projection datasets (10, 11))*						Constrained	-	2,426-2,336	TgC/yr	Reduction in Cook-Patton et al. 2020 mitigation based on urban and cropland expansion scenarios
Walker	Potential biomass = 'closed forest'	4,016	(12)	(13)	(14)	*None*	(15)	*None*	Constrained	223	22,700	TgC	Potential biomass for AGB + BGB + SOC pools
	Potential biomass = 'closed forest' or 'open forest'	8,161	(12)	(13)	(14)	*None*	(15)	*None*		932	53,800	TgC	

Map	Forest Potential: Criteria description	Forest Potential: Global area (Mha)	Exclusions: Existing forest	Exclusions: Cropland	Exclusions: Pasture	Exclusions: Wetland	Exclusions: Urban	Exclusions: Other	Restoration Potential: Type	Restoration Potential: Global area (Mha)	Restoration Potential: Global mitigation	Restoration Potential: Mitigation unit	Restoration Potential: Mitigation comment
This analysis	> 60% potential tree cover (Bastin) AND potential biomass = “woody systems” (Walker) AND no high uncertainty in Bastin or Walker models AND fire frequency < 2 for the period 2002-2022	2,242	(16)	*None*	*None*	*None*	*None*	Open water, bare ground, ice/snow	Maximum	305	849	TgCe/yr	Modeled annual natural forest regrowth rates over 30 years for AGB + BGB pools. Includes albedo offset, thus value is net mitigation potential. Note that units presented in the main paper are TgCO2e/yr.
	> 60% potential tree cover (Bastin) AND potential biomass = ‘closed forest’ (Walker) AND no high uncertainty in Bastin or Walker models AND fire frequency < 2 for the period 2002-2022	2,242	(16)	(17)	*None*	(18)	(19)	Albedo, open water, bare ground, ice/snow	Constrained	195	607	TgCe/yr	Modeled annual natural forest regrowth rates over 30 years for AGB + BGB pools. Includes albedo offset, thus value is net mitigation potential. Note that units presented in the main paper are TgCO2e/yr.
			(16)	(17)	*None*	(18)	(19)	Albedo, open water, bare ground, ice/snow	Constrained with additionality deduction	169	569	TgCe/yr	
(Sensitivity tests)			(20)	(17)	*None*	(18)	(19)	Albedo, open water, bare ground, ice/snow	Constrained	199	662	TgCe/yr	
			(16)	(21)	*None*	(18)	(19)	Albedo, open water, bare ground, ice/snow	Constrained	208	644	TgCe/yr	
	> 60% potential tree cover (Bastin) AND potential biomass = ‘closed forest’ (Walker) AND no high uncertainty in Bastin or Walker models	2,393	(16)	(17)	*None*	(18)	(19)	Albedo, open water, bare ground, ice/snow	Constrained	242	825	TgCe/yr	

Map	Forest Potential		Exclusions						Restoration Potential				
	Criteria description	Global area (Mha)	Existing forest	Cropland	Pasture	Wetland	Urban	Other	Type	Global area (Mha)	Global mitigation	Mitigation unit	Mitigation comment
(Sensitivity tests, continued)	> 50% potential tree cover (Bastin) AND potential biomass = 'closed forest' (Walker) AND no high uncertainty in Bastin or Walker models AND fire frequency < 2 for the period 2002-2022	2,675	(16)	(17)	*None*	(18)	(19)	Albedo, open water, bare ground, ice/snow	Constrained	265	747	TgCe/yr	Modeled annual natural forest regrowth rates over 30 years for AGB + BGB pools. Includes albedo offset, thus value is net mitigation potential. Note that units presented in the main paper are TgCO2e/yr.
	> 30% potential tree cover (Bastin) AND potential biomass = 'closed forest' or 'open forest' (Walker) AND fire frequency < 2 for the period 2002-2022	5,095	(16)	(17)	*None*	(18)	(19)	Albedo, open water, bare ground, ice/snow	Constrained	845	1,695	TgCe/yr	
	> 30% potential tree cover (Bastin) AND potential biomass = 'closed forest' or 'open forest' (Walker)	5,788	(16)	(17)	*None*	(18)	(19)	Albedo, open water, bare ground, ice/snow	Constrained	1,135	2,588	TgCe/yr	

(1) 2009 forest extent with 250m resolution (Cited as "Peter Potapov, Matthew Hansen, and Svetlana Turubanova. 2011. Global forest extent derived using MODIS 250m data. University of Maryland" at https://www.wri.org/data/methodology-data-atlas-forest-landscape-restoration-opportunity)
(2) 2008 cropland extent with 250m resolution [40]
(3) Excluded from forest suitability at biomes scale
(4) 2005 population estimate at 1km resolution [41]
(5) 2012 tree cover map with 30m resolution [42], forest defined as > 25% tree cover
(6) 2012 tree cover map with 30m resolution [42], forest defined as > 10% tree cover
(7) 2009 land cover map with 300m resolution (GlobCover), cropland types include mosaic classes ("mosaic crop (50-70%) and natural vegetation" and "mosaic vegetation and crop (20-50%)")
(8) 2005 cropland percentage map at 1km resolution [43]
(9) 2015 land cover map with 300m resolution (ESA CCI)
(10) 2010-2100 land use change [44]
(11) 2010-2100 urban expansion [45]
(12) 2016 aboveground carbon map at 500m resolution derived from [46], forest definition based on ratio of existing:potential biomass, where forest suitability pixels with E:P >0.25 are considered forested
(13) 2017 cropland map with 30m resolution, aggregated by authors to 500m using "majority-rule approach"
(14) 2000 fractional pasture cover map with 10km resolution [47], pasture defined as >50% fractional cover
(15) 2015 Global Human Settlement Layer, urban defined as > 300 people/km2
(16) 2020 forest extent [48], forest definition based on height (>= 5 m) and canopy criteria (>20-25%)
(17) 2020 cropland agreement map at 30m resolution [49], croplands defined as agreement in 3 or more of 6 maps is
(18) 2020 wetland map at 30m resolution [50] and global peatland map at 9km resolution [51]

(19) 2020 built lands map at 30m resolution [52]
(20) 2019-2020 forest extent [53], forest definition based on > 10% tree cover
(21) 2015, 2019 cropland map at 30m resolution [54]

Supplementary Table 3 | Exclusion and overlay datasets used in this analysis. This table outlines the datasets used in this analysis, their source, their spatial resolution, and their use, along with a description of any processing steps used to prepare the datasets.

Dataset	Source	Resolution	Description and processing steps	Use
Existing forest	GLAD Forest Extent and Height Change, 2000-2020 [52]	30 meters	Forest defined based on height (>= 5 m) and canopy criteria (>20-25%) for the years 2000 and 2020. Value set to 1 if 'stable', 'gain', or forest in both years with 'stand-replacement disturbances or degradation'; not masked if 'loss'. Multi-year mapping allows for accounting of regrowth.	Exclusion (implementation)
Existing forest (alternative)	Global 4-class PALSAR-2/PALSAR Forest/Non-Forest Map 2019-2020 [53]	25 meters	Forest defined based on area (> 0.5 ha) and tree cover (>10%) for the years 2019 and 2020. Value set to 1 if classified as 'dense forest' or 'non-dense forest'. Dataset resampled to 30m using nearest neighbor.	Sensitivity test: exclusion (implementation)
Open water	GLAD surface water 1999-2020 [55]	30 meters	Values set to 1 for pixels with 'permanent water', 'stable seasonal', 'water gain', 'water loss', 'dry period', 'wet period, 'high frequency', or 'probable permanent water' classes for 1999-2020.	Exclusion (implementation)
Bare ground	ESA WorldCover 10m v100 and v200 [56,57]	10 meters	Bare ground = "Lands with exposed soil, sand or rocks and never has more than 10% vegetated cover during any time of the year." Value set to 1 when bare ground is mapped in 2020 and 2021. Multi-year mapping ensures short-term disturbances (e.g., logging, wildfire) are not masked. Dataset resampled to 30m using nearest neighbor.	Exclusion (implementation)
Ice/snow	GLAD Perennial Snow and Ice Extent and Change, 2000-2020 [52]	30 meters	Values set to 1 for pixels with perennial snow and ice from 2000-2020.	Exclusion (implementation)
Built-up lands	GLAD Built-up Lands Extent and Change, 2000-2020 [52]	30 meters	Built-up defined as "man-made land surfaces associated with infrastructure, commercial and residential land uses." Value set to 1 if classified as 'stable' or 'expansion' for 2020.	Exclusion (precautionary)
Peatlands	Peat-ML [51]	5 kilometers	Identifies areas with peatlands based on climate and soil factors and remotely-sensed vegetation indices. Values set to 1 if > 50% predicted peatland cover. Dataset resampled to 30m using nearest neighbor.	Exclusion (precautionary)

Dataset	Source	Resolution	Description and processing steps	Use
Wetlands	GWL_FSC30 Wetlands 2020 [50]	30 meters	Values set to 1 for pixels with 'permanent water', 'swamp', 'marsh', 'flooded flat', 'saline', 'mangrove', 'salt marsh', or 'tidal flat' classes for the year 2020.	Exclusion (precautionary)
Cropland	Global cropland map agreement 2020 [49]	30 meters	Provides pixel-scale count of agreement of 6 cropland maps from 2020; source maps use various definitions of croplands, including annual and permanent herbaceous and woody crops for human consumption, forage, and biofuel. Value set to 1 where cropland map agreement count is 3 or more.	Exclusion (precautionary)
Cropland (alternative)	GLAD cropland 2015, 2019 [54]	30 meters	Cropland definition includes land used for annual and perennial herbaceous crops for human consumption, forage (including hay), and biofuel and excludes perennial woody crops, permanent pastures, and shifting cultivation. Value set to 1 where cropland occurs in 2015 or 2019. Multi-year mapping allows for accounting of fallow lengths up to four years.	Sensitivity test: exclusion (precautionary)
Albedo	Hasler et al. albedo [58]	0.005 degrees	Estimates the net climate impact of converting non-forest LULC to forest. Values set to 1 for pixels with net impact < 0 (net warming). Dataset resampled to 30m using nearest neighbor.<br><br>Also applied to mitigation estimate as offset (TgCe per ha per yr).	Exclusion (precautionary),<br><br>Overlay
Climate change mitigation (carbon sequest-ration)	Cook-Patton et al. natural regrowth sequestration [59]	1 kilometer	Estimates the rate of aboveground and belowground carbon sequestration during the first 30 years of natural regrowth. Values are TgCe per ha per year.	Overlay
Population	Gridded Population of World Version 4 (GPWv4) [60]	1 kilometer	Estimates 2020 per-pixel population	Overlay
Background forest	GLAD Forest Extent and Height Change, 2000-2020 [52]	30 meter, summarized to 1 degree	Maps forest loss year and binary gain for the period 2000-2020. Loss:gain ratio calculated as the total area with forest loss divided by total area with forest gain per 1x1 degree cell. Used to estimate the proportion of	Overlay

Dataset	Source	Resolution	Description and processing steps	Use
loss:gain ratio			constrained reforestation potential (area or mitigation estimate) where restoration would exceed business as usual forest recovery.	
High individual and minority rights	Varieties of Democracy (V-Dem) Project [61]	National	Liberal democracy index values reflect how individuals and local communities can influence decision making, as indicated by constitutionally protected civil liberties, strong rule of law, an independent judiciary, and effective checks and balances to government and/or majority power. Values set to 1 for countries with liberal democracy index values for 2023 that are above the global median (0.362). National scale vector data converted to raster format with 500m resolution.	Overlay in high individual rights scenario
Secure land tenure	LandMark Percentage of Indigenous and Community Lands Not Recognized by Governments and Legal Status of Indigenous and Community Lands, 2021 [62] and Prindex Tenure Insecurity, 2020 [63]	National	A composite measure derived from multiple land tenure datasets from the period 2020-2021 (datasets are combined in order to minimize the effect of missing data ('no data') in source datasets). See Table S4 for additional details. Any remaining countries with missing data are assigned a value of 0. National scale vector data converted to raster format with 500m resolution.	Overlay in secure land tenure scenario
Secure land tenure (alternative #1)	LandMark Percentage of Indigenous and Community Lands Not Recognized by Governments, 2021 [62]	National	Indicates the percentage of indigenous and community lands not recognized by governments in 2021. Values set to 1 for countries with < 20% indigenous and community lands not recognized by governments. Note that missing data ('no data') is assigned value of 0. National scale vector data converted to raster format with 500m resolution.	Overlay in secure land tenure scenario and sensitivity test
Secure land tenure (alternative #2)	LandMark Legal Status of Indigenous and Community Lands, 2021 [62]	National	A composite measure based on 10 indicators (legal status, land rights and common property, formal documentation, legal person, legal authority, perpetuity, right to consent before land acquisition, rights to trees, rights to water, land rights in protected areas) in 2021 scored on a 1 – 4 scale (1 – yes, the law fully addresses the issue to 4 – no, the law does not address the issue). Values set to 1 for countries where the legal security of Indigenous lands is less than or equal to the global average (2.8). Note that missing data ('no data') is assigned value of 0. National scale vector data converted to raster format with 500m resolution.	Overlay in secure land tenure scenario and sensitivity test

Dataset	Source	Resolution	Description and processing steps	Use
Secure land tenure (alternative #3)	LandMark Legal Status of Indigenous and Community Lands, 2021 [62]	National	A composite measure based on 10 indicators (legal status, land rights and common property, formal documentation, legal person, legal authority, perpetuity, right to consent before land acquisition, rights to trees, rights to water, land rights in protected areas) in 2021 scored on a 1 – 4 scale (1 – yes, the law fully addresses the issue to 4 – no, the law does not address the issue). Values set to 1 for countries where the legal security of community lands is less than or equal to the global average (2.8). Note that missing data ('no data') is assigned value of 0. National scale vector data converted to raster format with 500m resolution.	Overlay in secure land tenure scenario and sensitivity test
Secure land tenure (alternative #4)	Prindex Tenure Insecurity, 2020 [63]	National	Indicates percentage of survey respondents in 2020 who believe it is "somewhat or very likely that they could lose the right to use their property or part of it against their will in the next 5 years." Values set to 1 for countries with < 20% of respondents reporting tenure insecurity. Note that missing data ('no data') is assigned value of 0. National scale vector data converted to raster format with 500m resolution.	Overlay in secure land tenure scenario and sensitivity test
Vulnerable rural livelihoods	Global Relative Deprivation Index v1 2010 [64], Global Friction Surface [65]	500 meters	Uses the Global Relative Deprivation Index (GRDI), which is based on childhood dependency ratios, infant mortality, human well-being, and economic development measures for the year 2010, and a global walking travel time map. Values set to 1 for pixels within a 1 hour walk of areas with GRDI values less than the highest quartile (77.25).	Overlay in low rural livelihood conflict scenario
≥ 30% forest within 5 km	GLAD Forest Extent and Height Change, 2000-2020 [52]	30 meters	Percent forest within 5 km radius using 30m existing forest dataset for the years 2000 and 2020. Values set to 1 for pixels at least 30% forest within 5km. See description of 'existing forest' dataset above for additional information. Dataset resampled to 1km using nearest neighbor.	Overlay in forest neighborhood scenario
Moderate slopes	NASA SRTM Digital Elevation Data Version 4	90 meters	Slope as percent. Values set to 1 where slopes 20-50%.	Overlay in water quality scenario
Floodplains	Global ALOS landforms [66]	90 meters	Landform type. Values set to 1 where landform type is 'Lower slope (flat)', 'Valley', or 'Valley (narrow)'	Overlay in water quality scenario

Dataset	Source	Resolution	Description and processing steps	Use
Forest restoration goals	Goals and Commitments for the Restoration Decade [67]	National	Identifies countries that have committed to restoring or increasing forest through Land Degradation Neutrality, National Biodiversity Strategies and Action Plans, Nationally Determined Contributions (Paris Agreement), or Bonn Challenge pledges as of 2020. National scale vector data converted to raster format with 500m resolution.	Overlay in national forest restoration goal scenario
Existing protected areas	World Database on Protected Areas [68]	NA	Protected areas in 2023. Values set to 1 where IUCN categories are Ia, Ib, II, or III (management focus on conservation/protection, not multiple uses). National scale vector data converted to raster format with 500m resolution.	Overlay in protected areas scenario

Supplementary Table 4 | Area of intersection (agreement) for this analysis and other global forest potential and maximum or constrained reforestation potential maps. This table lists the components of the unique global reforestation maps and reports the area of intersection (agreement) with various components of this analysis.

Other global restoration map			Area of intersection (agreement) with this analysis (million hectares)				
**Name**	**Component**	**Area (Mha)**	**Forest potential (total area: 2,242 Mha)**	**Forest suitability criteria (<2 non-crop fires 2002-2022 @1km scale)**	**Exclusions: imple-mentation constraints**	**Exclusions: pre-cautionary constraints**	**Constrained reforestation potential (total area: 195 Mha)**
FLRO	Forest potential: > 45% potential canopy cover	4,253	2,051	3,980	-	-	-
	Forest potential: > 25% potential canopy cover	5,706	2,192	5,101	-	-	-
	Forest potential: > 10% potential canopy cover	7,506	2,233	6,524	-	-	-
	Constrained reforestation potential: > 45% canopy cover ('Wide-scale restoration')	514	23	510	75	65	5
	Constrained reforestation potential: > 10% canopy cover ('Wide-scale, remote, and mosaic restoration')	2,509	388	1,981	884	750	98
Griscom	Forest potential: (FLRO > 25% canopy cover)	5,706	2,192	5,101	-	-	-
	Constrained reforestation potential ('Reforestation' before deductions for background regrowth and other NCS pathways)	852	208	786	346	239	56
Bastin	Forest potential: >10% potential tree cover	8,682	2,242	7,624	-	-	-
	Select exclusion: Existing forest (> 10% tree cover)	4,881	2,043	-	3,709	350	132
	Select exclusion: Globcover croplands	2,823	322	-	593	1,700	59
	Constrained reforestation potential ('Forest cover restoration' with Globcover exclusions)	1,772	92	1,564	302	936	32
Strass-burg	Forest potential: > 15% tree cover in predominant 'original ecosystem' type	6,160	2,146	5,534	-	-	-
	Maximum reforestation potential	1,537	279	1,469	450	786	61
Walker	Forest potential: Potential biomass = 'closed forest'	4,016	2,242	3,565	-	-	-
	Forest potential: Potential biomass = 'closed forest' or 'open forest'	8,161	2,242	7,124	-	-	-
	Select exclusion: existing forest (current:potential biomass ratio >0.25)	6,097	2,112	-	3,965	912	153
	Select exclusion: "societal constraints" (cropland, shifting agriculture, grazing lands, and urban areas)	4,195	213	-	834	2,540	50

Other global restoration map			Area of intersection (agreement) with this analysis (million hectares)				
**Name**	**Component**	**Area (Mha)**	**Forest potential (total area: 2,242 Mha)**	**Forest suitability criteria (<2 non-crop fires 2002-2022 @1km scale)**	**Exclusions: imple-mentation constraints**	**Exclusions: pre-cautionary constraints**	**Constrained reforestation potential (total area: 195 Mha)**
Walker, cont.	Constrained reforestation potential (‘Restore, high suitability for forestry-based natural climate solutions’)	223	51	194	53	54	24
	Constrained reforestation potential (‘Restore, high suitability for forestry-based natural climate solutions’ and ‘Restore, low suitability for forestry-based natural climate solutions’	932	53	818	149	412	25

Supplementary Table 5 | Composite secure land tenure overlay data sources and sensitivity analysis results. This table outlines the individual land tenure datasets, how those datasets were applied for sensitivity testing, and describes the composite land tenure measure used in the land tenure scenario. All land tenure data are available at the Level 0 administrative unit scale.

Dataset	Application	# of Level 0 admin. units with data	% of global land area with data	Scen-ario area (Mha)	Scenario mitigation potential ($TgCO_2e/yr$)	Popula-tion impacted (million)
LandMark Percentage of Indigenous and Community Lands Not Recognized by Governments, 2021 [62]	A measure of land tenure security based on the percentage of indigenous and community lands not recognized by governments in 2021. A country is considered to have secure land tenure when < 20% Indigenous and community lands are not recognized by country governments, following [69].	98	72.3%	75	1,181	32
LandMark Legal Status of Indigenous Lands, 2021 [62]	A measure of land tenure security based on 10 indicators (legal status, land rights and common property, formal documentation, legal person, legal authority, perpetuity, right to consent before land acquisition, rights to trees, rights to water, land rights in protected areas) in 2021 scored on a 1 – 4 scale (1 – yes, the law fully addresses the issue to 4 – no, the law does not address the issue). A country is considered to have secure land tenure when the legal security of Indigenous lands is ≤ 2.8 (global average).	91	85.3%	72	1,296	39
LandMark Legal Status of Community Lands, 2021 [62]	A measure of land tenure security based on 10 indicators (legal status, land rights and common property, formal documentation, legal person, legal authority, perpetuity, right to consent before land acquisition, rights to trees, rights to water, land rights in protected areas) in 2021 scored on a 1 – 4 scale (1 – yes, the law fully addresses the issue to 4 – no, the law does not address the issue). A country is considered to have secure land tenure when the legal security of community lands is ≤ 2.8 (global average).	95	51.7%	125	1,730	55
Prindex Tenure Insecurity, 2020 [63]	A measure of land tenure security based on the percentage of survey respondents in 2020 who believe it is "somewhat or very likely that they could lose the right to use their property or part of it against their will in the next 5 years." A country is considered to have secure land tenure when < 20% of respondents reporting tenure insecurity.	136	90.5%	109	553	42

Dataset	Application	# of Level 0 admin. units with data	% of global land area with data	Scen-ario area (Mha)	Scenario mitigation potential ($TgCO_2e/yr$)	Popula-tion impacted (million)
Composite measure	Uses LandMark Percentage of Indigenous and Community Lands Not Recognized by Governments in 2021, LandMark Legal Status of Indigenous Lands in 2021, LandMark Legal Status of Community Lands in 2021, and Prindex Tenure Insecurity measures for 2020 to identify countries with secure land tenure. Countries are classified for land tenure security following the classification rules below; the composite measure is based on the majority classification. Missing data are ignored for majority assignment. Countries with single measures use that value. If there is not a majority, a country is not considered to have secure land tenure.	173	99.0%	116	1,428	47

Supplementary Figure 1: Reforestation map coverage.

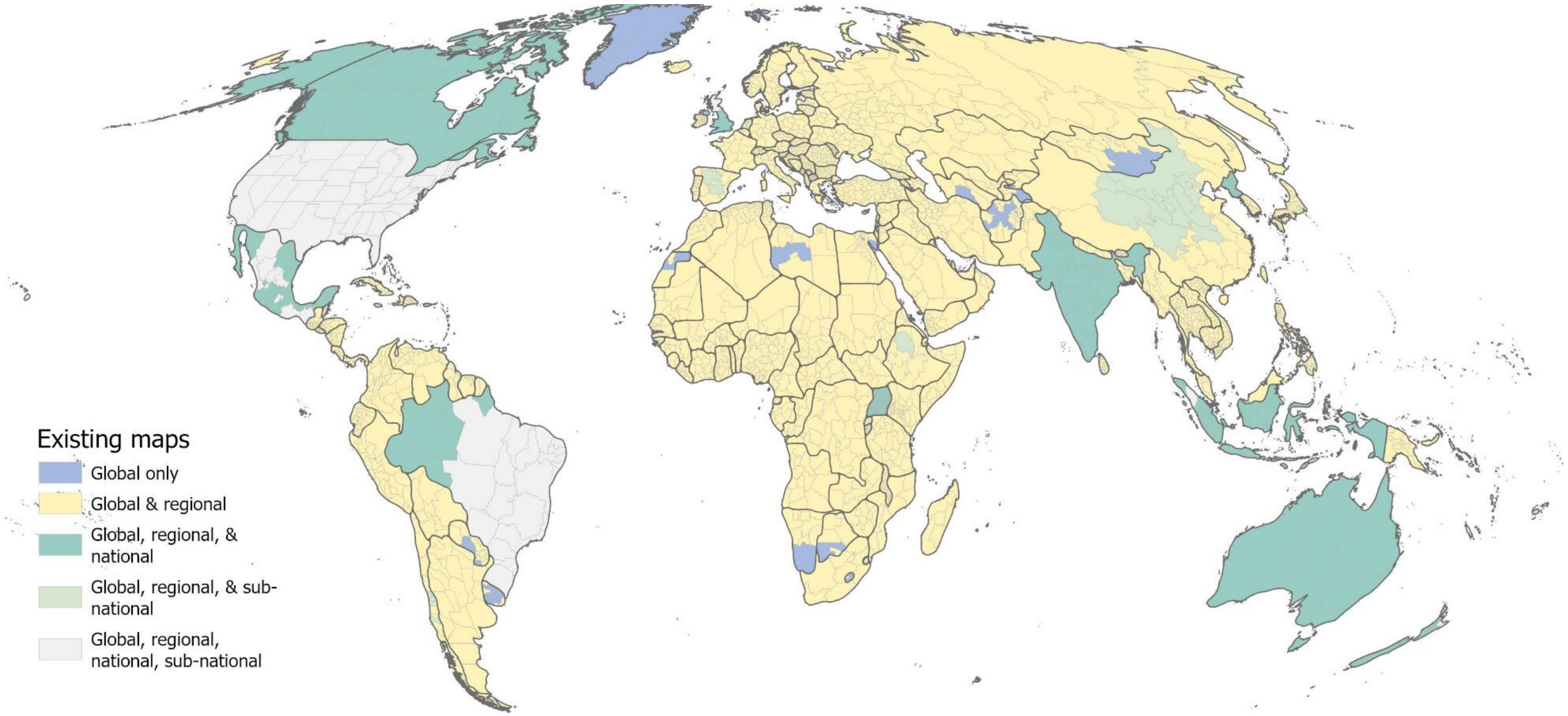

Coverage of peer-reviewed reforestation maps published 2011-2022 (n = 89) by Global Administrative Areas v 4.1 level 1 unit.

Supplementary Figure 2: Forest potential map agreement.

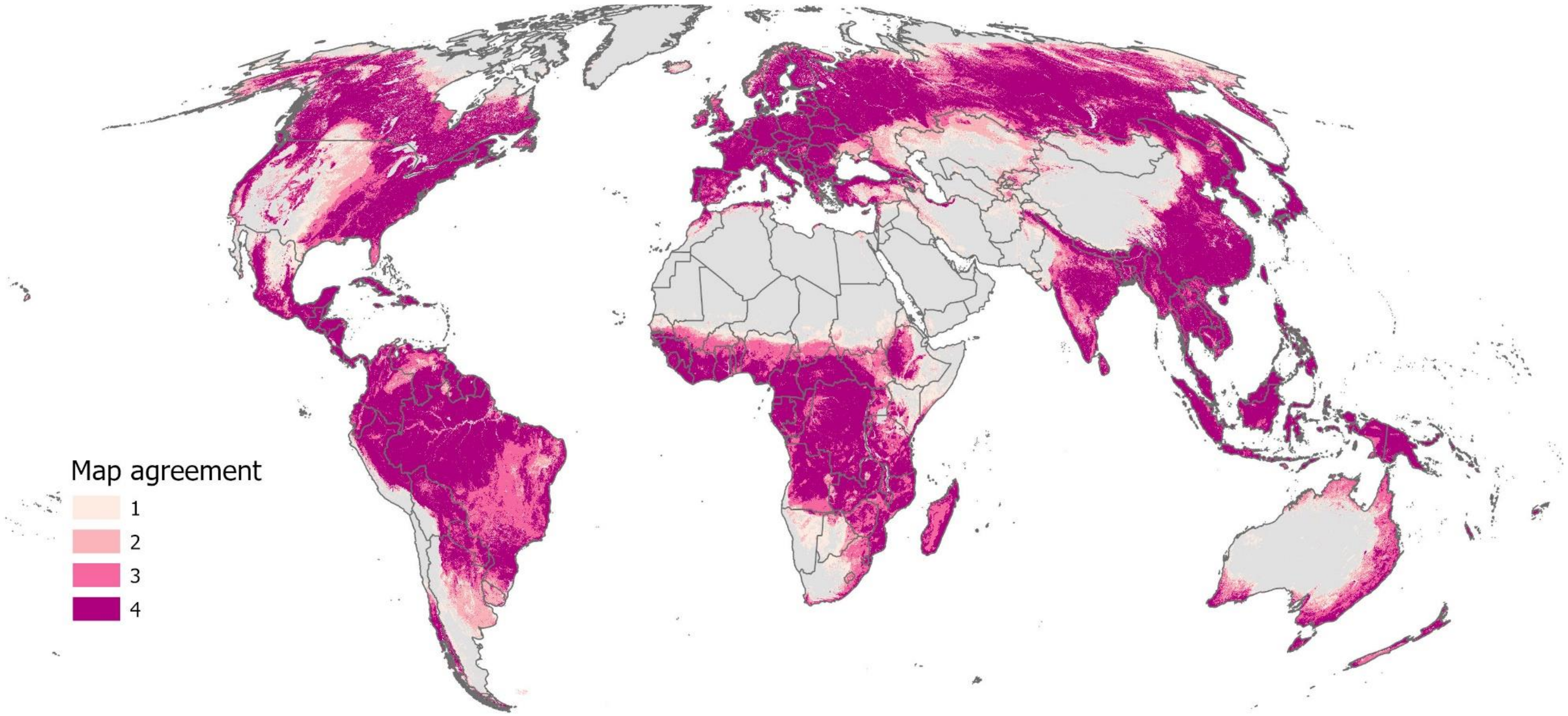

Count of agreement of the FLRO, Bastin, Strassburg, and Walker forest potential maps using the definitions of forest in the source products.

Supplementary Figure 3: Constrained reforestation potential map agreement.

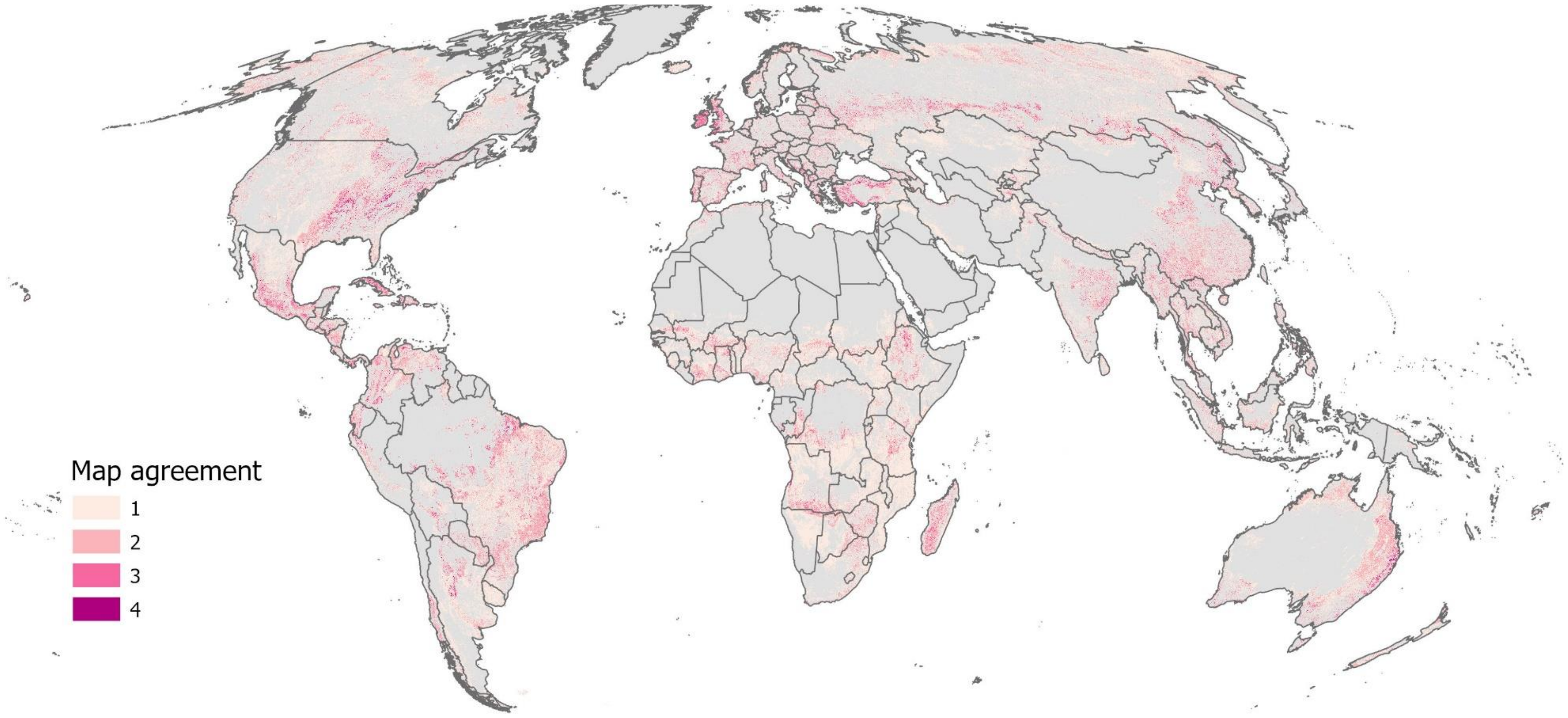

Count of agreement of the FLRO, Griscom, Bastin, and Walker constrained reforestation potential maps.

Supplementary Figure 4: Forest potential map.

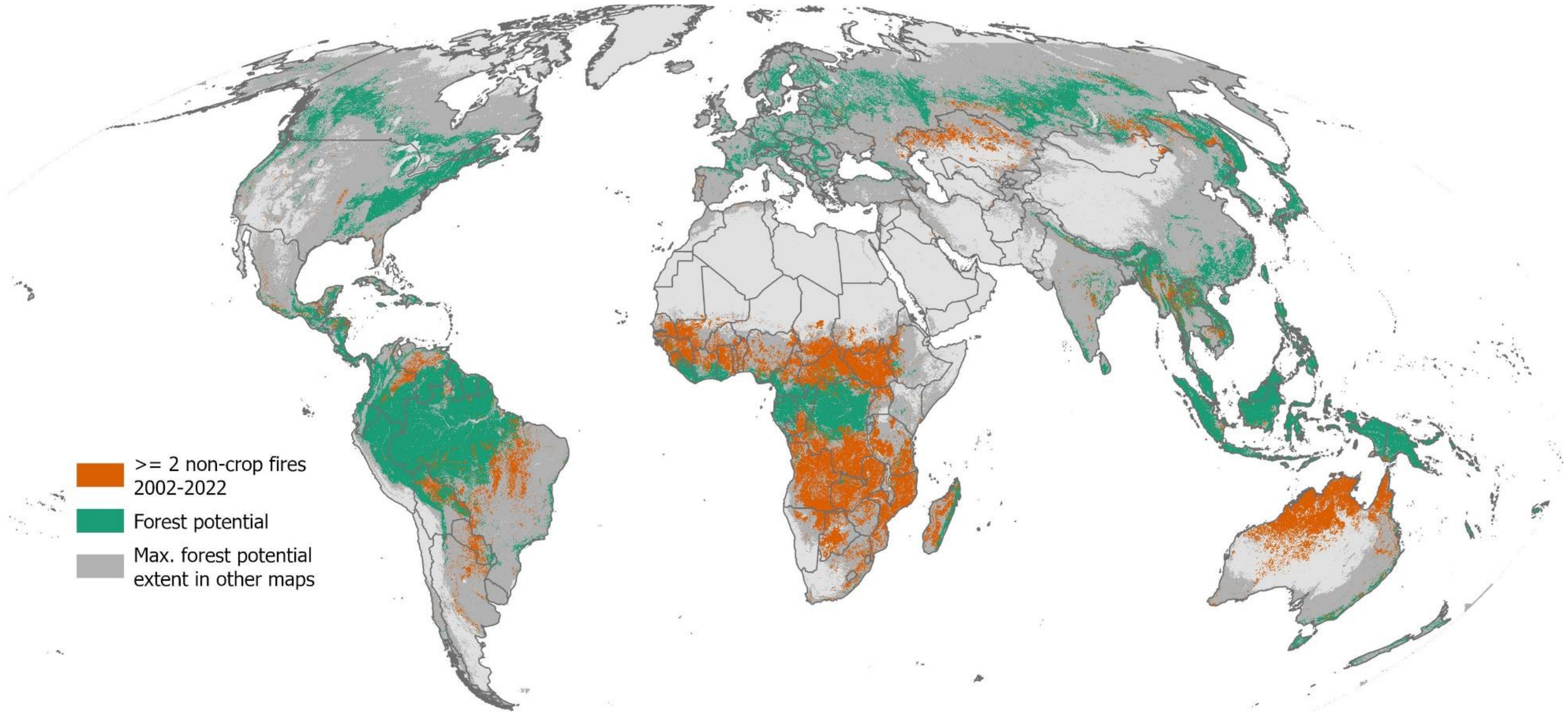

The forest potential map generated by this study, areas with at least 2 non-cropland fires during 2002-2022, and the maximum extent of forest potential from previously published maps.

Supplementary Figure 5: Forest loss:gain ratio, 2000-2020.

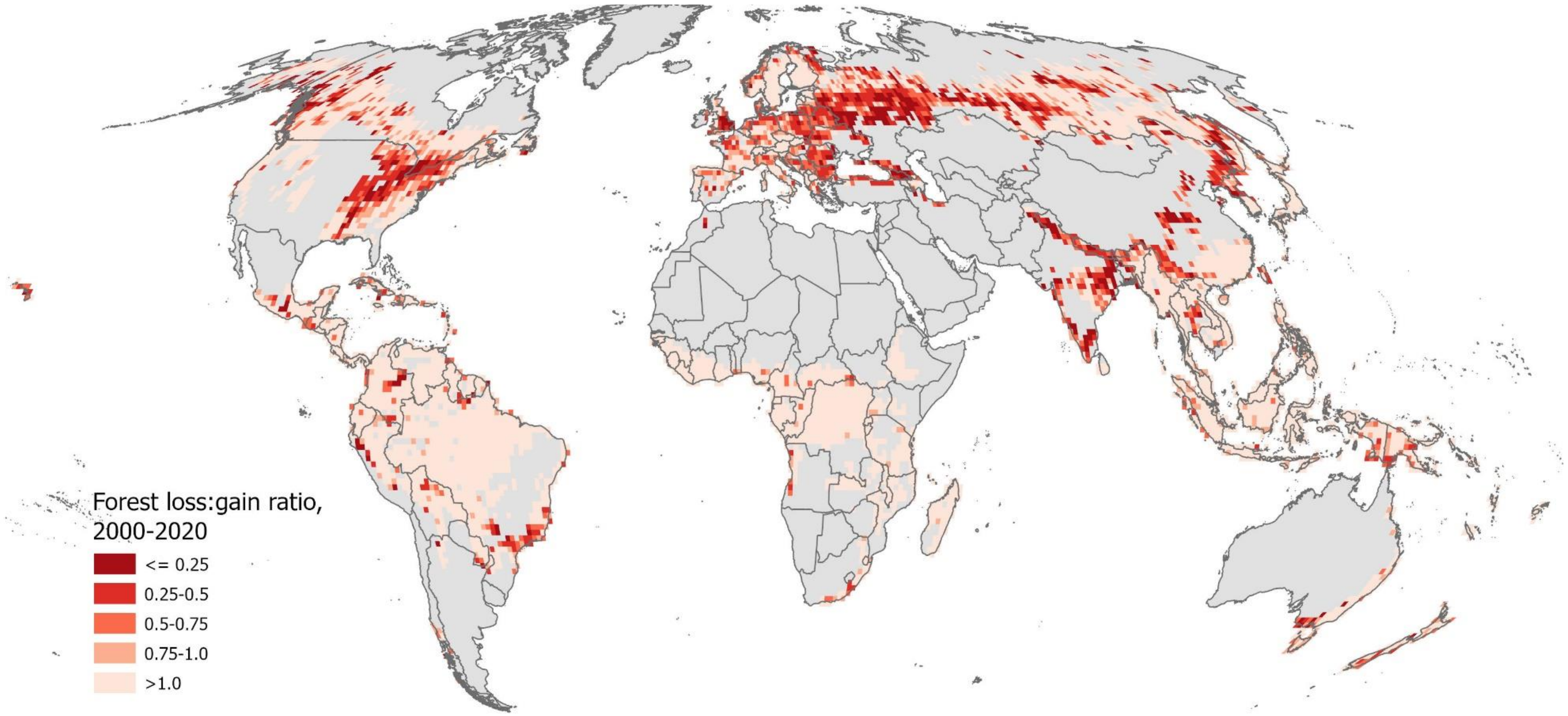

Ratio of the area of forest loss to forest gain for 2000-2020 by one-degree cell. These values can be interpreted to show the proportion of area or mitigation per cell where reforestation may be additional.

Supplementary Figure 6: Avoiding social conflicts scenarios.

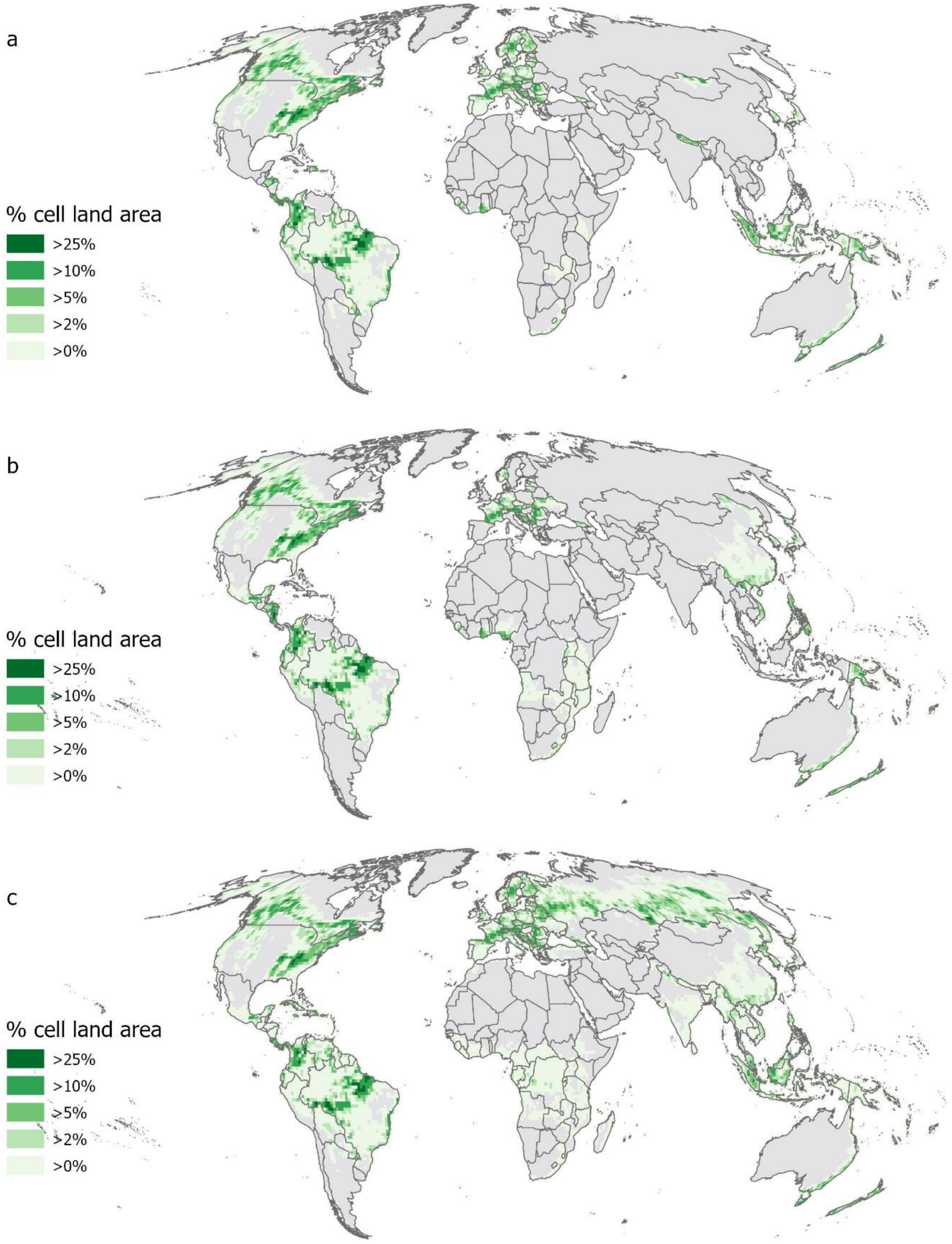

Percent land area with constrained reforestation potential in the (a) high individual rights scenario, (b) secure land tenure scenario, and (c) low rural livelihood conflict scenario. 1 km-scale data are aggregated by one-degree cell for display.

Supplementary Figure 7: Ecosystem services scenarios.

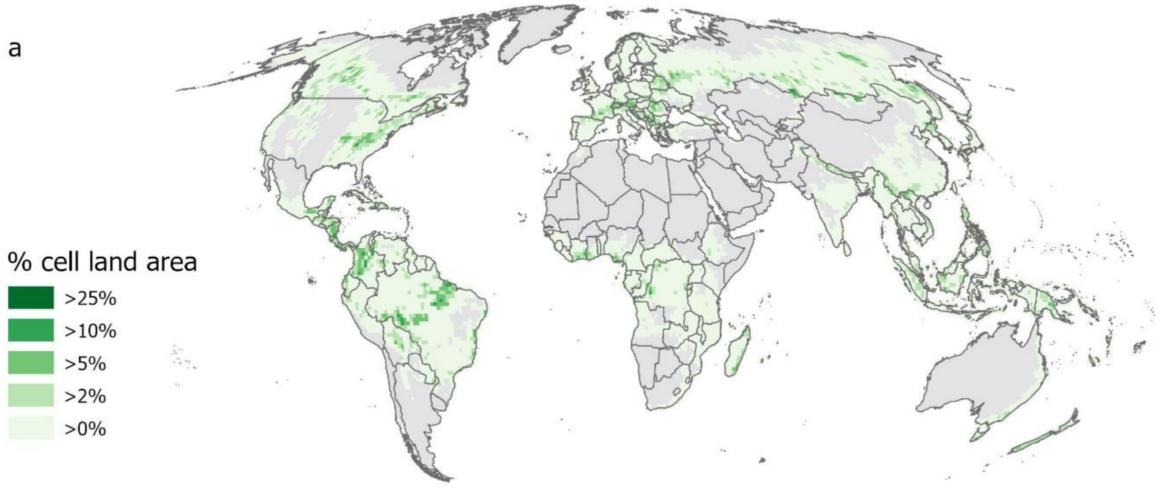

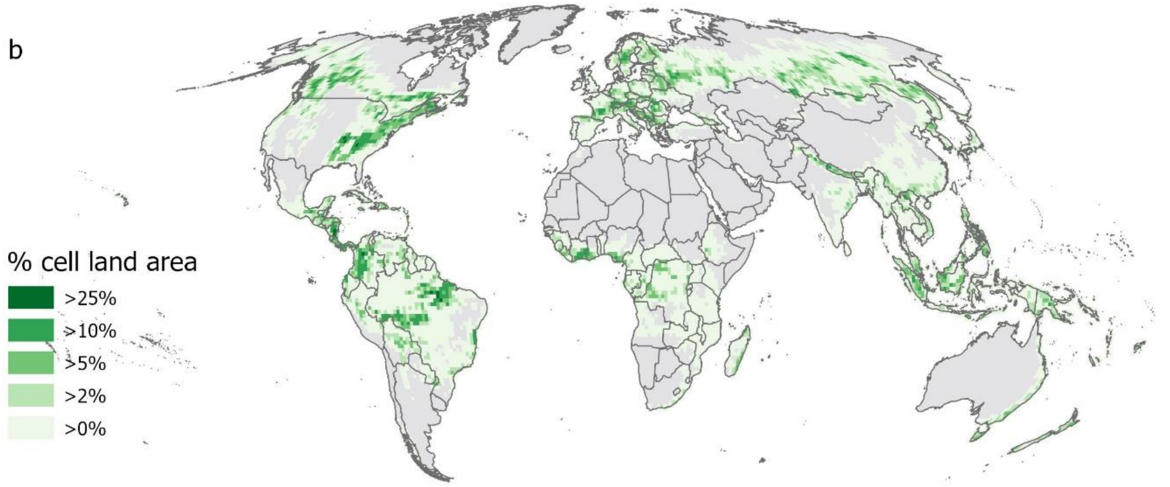

Percent land area with constrained reforestation potential in the (a) water quality scenario and (b) forest neighborhood scenario. 1 km-scale data are aggregated by one-degree cell for display.

Supplementary Figure 8: Government policy scenarios.

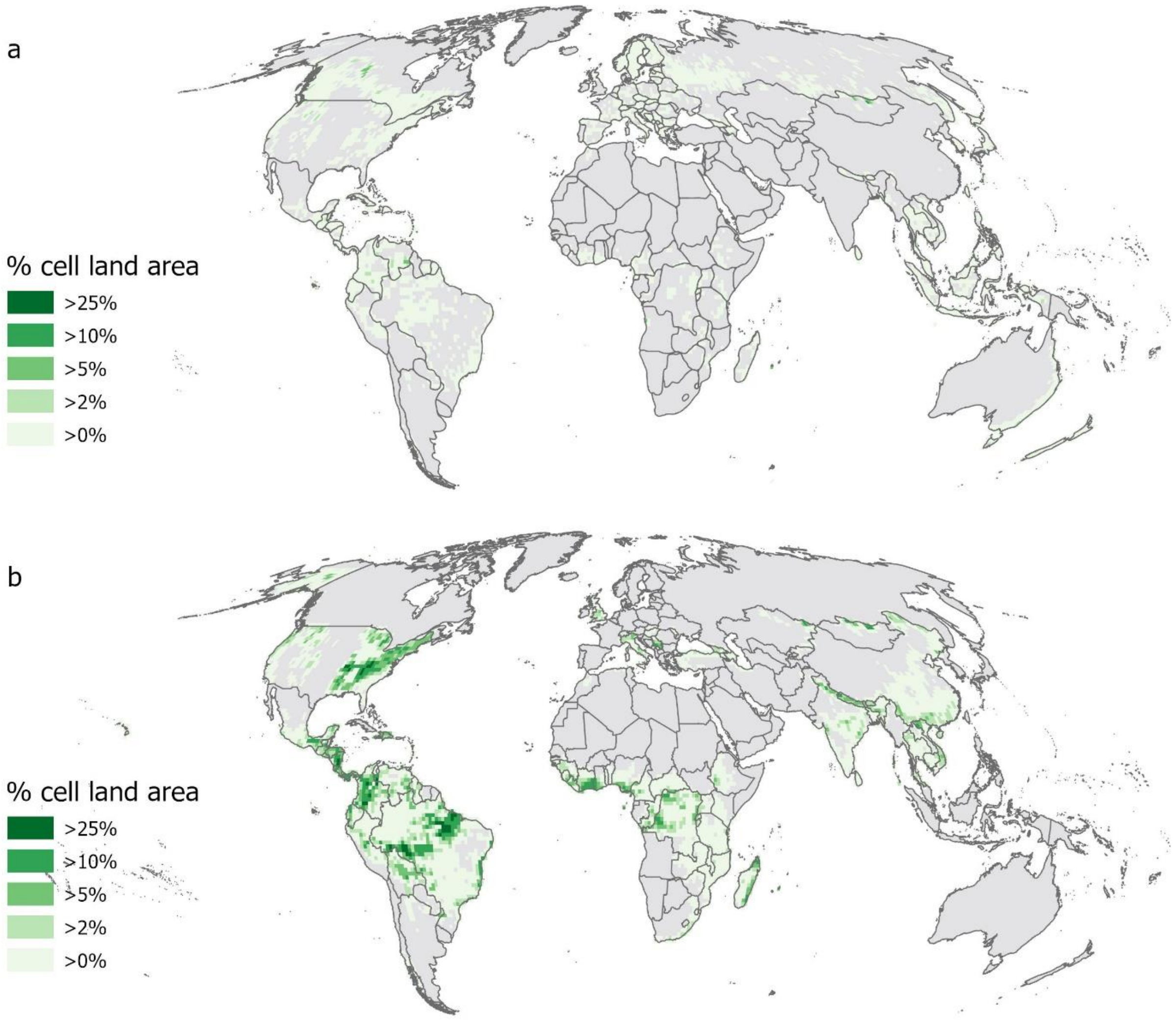

Percent land area with constrained reforestation potential in the (a) protected areas scenario and (b) national restoration goals scenario. 1 km-scale data are aggregated by one-degree cell for display.

Supplementary Figure 9: Sensitivity testing – forest potential.

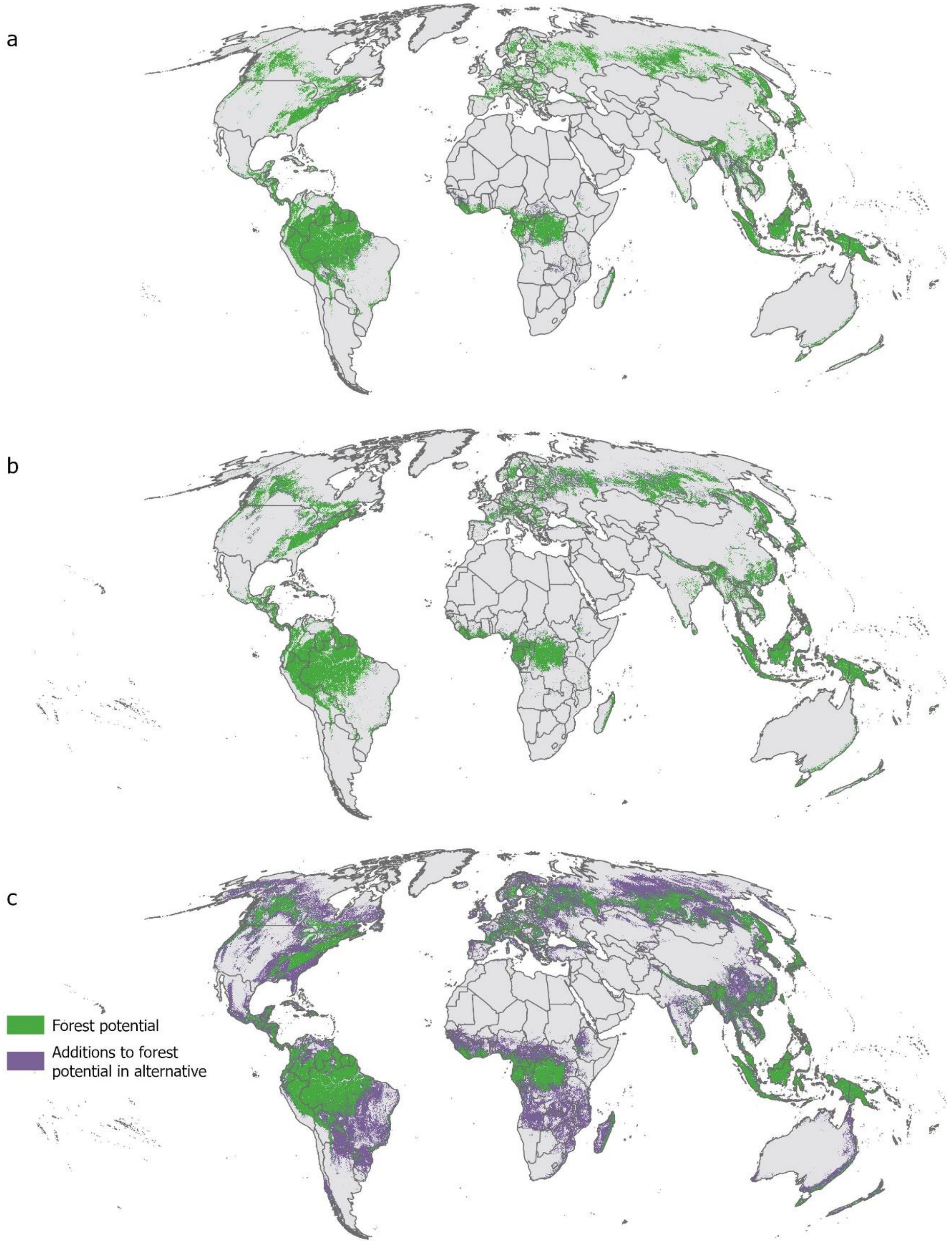

This study's forest potential map in comparison to alternatives: (a) no fire exclusions, (b) a 50% potential tree cover criterion, and (c) lower criteria for potential tree cover (>30%) and potential biomass (closed or open forest).

Supplementary Figure 10: Sensitivity testing - exclusions

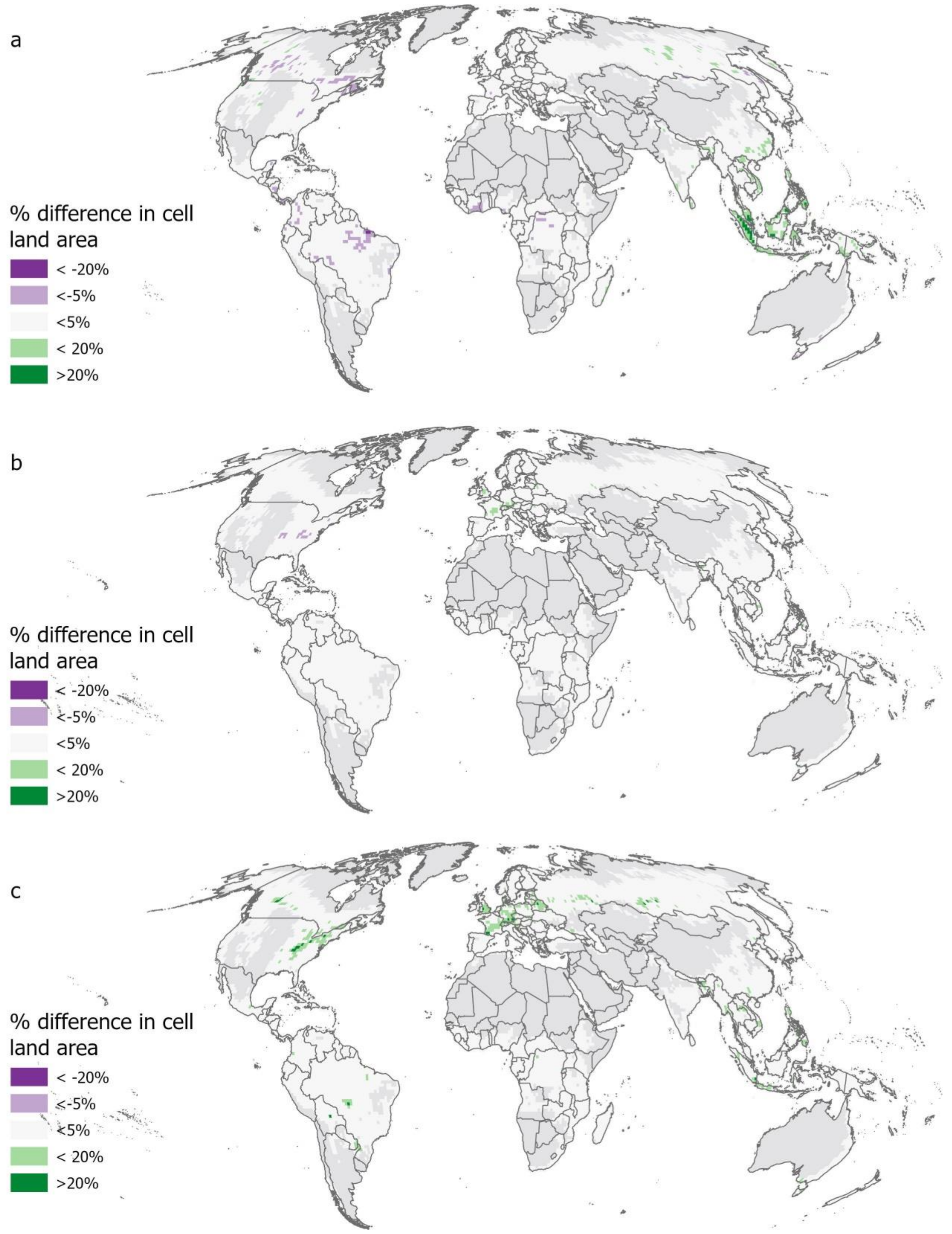

Percent difference in constrained reforestation potential with use of (a) an alternative product for excluding existing forest, (b) an alternative product for excluding croplands, and (c) without using croplands as an exclusion. 1 km-scale data are aggregated by one-degree cell for display.

Supplementary Figure 11: PRISMA diagram for literature review methods.

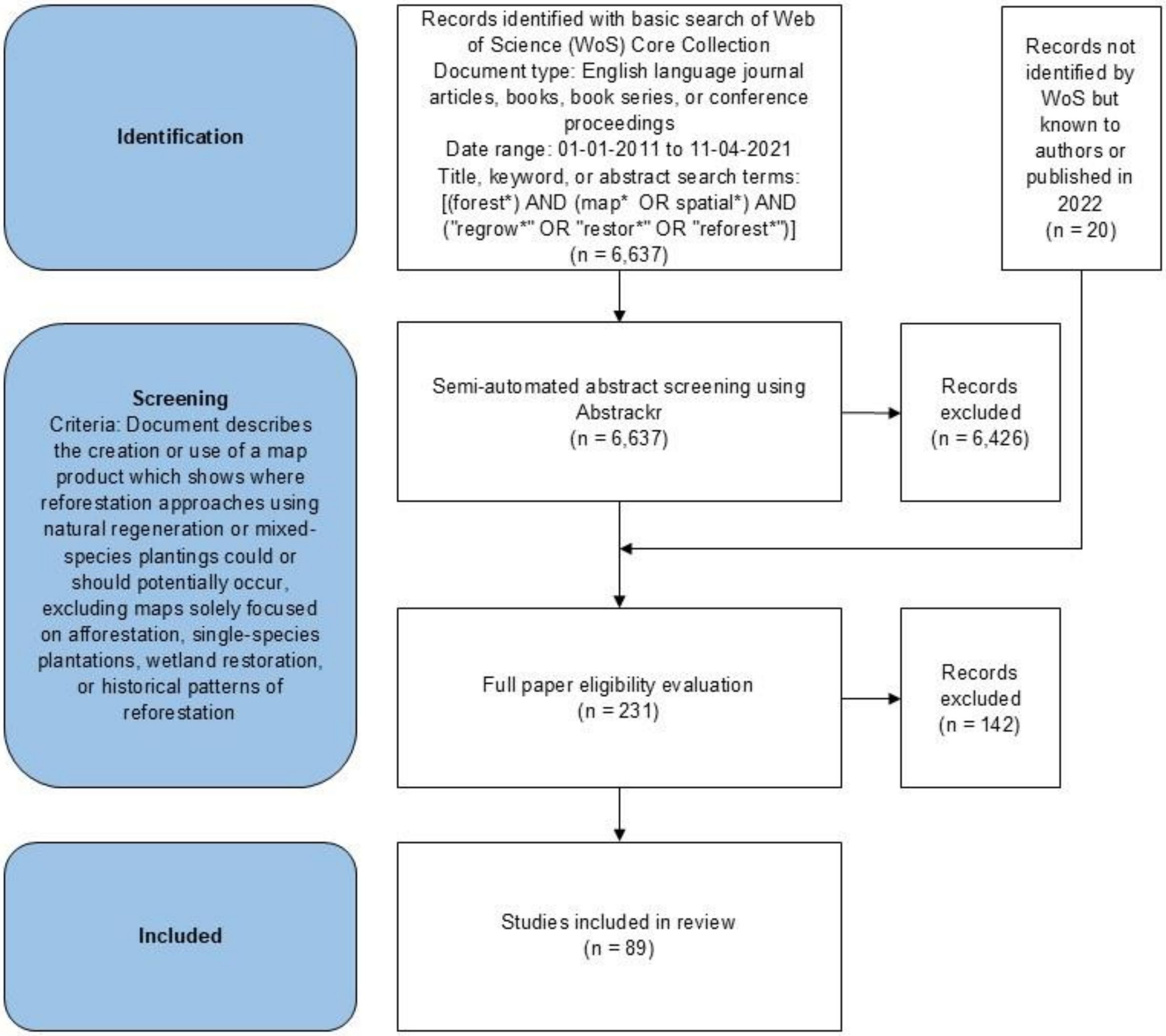

We conducted a literature to identify existing reforestation maps, broadly describe their geographic extent, and describe their application of spatial data. This figure provides an overview of the identification and screening methods and results used to identify the 89 previously published maps we reviewed.

Supplementary References

1. Keles, S., Günlü, A. & Baskent, E. Z. Identifying priority areas for reforestation using remote sensing and geographical information systems: a case study from Turkey. *Int J Global Warm* **5**, 109 (2013).

2. Erbaugh, J. T. *et al.* Global forest restoration and the importance of prioritizing local communities. *Nat Ecol Evol* **4**, 1472–1476 (2020).

3. Pascual, A., Giardina, C. P., Selmants, P. C., Laramee, L. J. & Asner, G. P. A new remote sensing-based carbon sequestration potential index (CSPI): A tool to support land carbon management. *Forest Ecol Manag* **494**, 119343 (2021).

4. Orsi, F., Church, R. L. & Geneletti, D. Restoring forest landscapes for biodiversity conservation and rural livelihoods: A spatial optimisation model. *Environ Modell Softw* **26**, 1622–1638 (2011).

5. Zeng, Y. *et al.* Economic and social constraints on reforestation for climate mitigation in Southeast Asia. *Nat Clim Change* **10**, 842–844 (2020).

6. Graves, R. A. *et al.* Potential greenhouse gas reductions from Natural Climate Solutions in Oregon, USA. *Plos One* **15**, e0230424 (2020).

7. Crouzeilles, R. *et al.* Achieving cost-effective landscape-scale forest restoration through targeted natural regeneration. *Conserv Lett* **13**, (2020).

8. Crouzeilles, R. *et al.* A new approach to map landscape variation in forest restoration success in tropical and temperate forest biomes. *J Appl Ecol* **56**, 2675–2686 (2019).

9. Fargione, J. E. *et al.* Natural climate solutions for the United States. *Sci Adv* **4**, eaat1869 (2018).

10. Gourevitch, J. D. *et al.* Optimizing investments in national-scale forest landscape restoration in Uganda to maximize multiple benefits. *Environ Res Lett* **11**, 114027 (2016).

11. Wilkes, M. A. *et al.* Making Way for Trees? Changes in Land-Use, Habitats and Protected Areas in Great Britain under "Global Tree Restoration Potential." *Sustainability-basel* **12**, 5845 (2020).

12. Echeverria, C., Gatica, P. & Fuentes, R. Habitat Edge Contrast as an Indicator to Prioritize Sites for Ecological Restoration at the Landscape Scale. *Natureza Conservação* **11**, 170–175 (2013).

13. Busch, J. *et al.* Potential for low-cost carbon dioxide removal through tropical reforestation. *Nat Clim Change* **9**, 463–466 (2019).

14. West, T. A. P. *et al.* Comparison of spatial modelling frameworks for the identification of future afforestation in New Zealand. *Landscape Urban Plan* **198**, 103780 (2020).

15. Uribe, D., Geneletti, D., Castillo, R. del & Orsi, F. Integrating Stakeholder Preferences and GIS-Based Multicriteria Analysis to Identify Forest Landscape Restoration Priorities. *Sustainability-basel* **6**, 935–951 (2014).

16. Syartinilia, Wahyuni, S., Rabbani, A., Idris, N. & Santoso, I. Ecological restoration planning based on multi-criteria approach in landscape of South Tapanuli Regency, Indonesia. *Sixth Int Symposium Lapan-ipb Satell* **11372**, 113721C-113721C–11 (2019).

17. Teo, H. C. *et al.* Global urban reforestation can be an important natural climate solution. *Environ Res Lett* **16**, 034059 (2021).

18. Gopalakrishna, T. *et al.* Existing land uses constrain climate change mitigation potential of forest restoration in India. *Conserv Lett* (2022) doi:10.1111/conl.12867.

19. Zheng, Q. *et al.* Future land-use competition constrains natural climate solutions. *Sci Total Environ* **838**, 156409 (2022).

20. Molin, P. G., Chazdon, R., Ferraz, S. F. de B. & Brancalion, P. H. S. A landscape approach for cost-effective large-scale forest restoration. *J Appl Ecol* **55**, 2767–2778 (2018).

21. Nunes, F. S. M., Soares-Filho, B. S., Rajão, R. & Merry, F. Enabling large-scale forest restoration in Minas Gerais state, Brazil. *Environ Res Lett* **12**, 044022 (2017).

22. Santos, J. S. dos *et al.* Characterising the spatial distribution of opportunities and constraints for land sparing in Brazil. *Sci Rep-uk* **10**, 1946 (2020).

23. Lemos, C. M. G., Andrade, P. R., Rodrigues, R. R., Hissa, L. & Aguiar, A. P. D. Combining regional to local restoration goals in the Brazilian Atlantic forest. *Reg Environ Change* **21**, 68 (2021).

24. Cook-Patton, S. C. *et al.* Lower cost and more feasible options to restore forest cover in the contiguous United States for climate mitigation. *One Earth* **3**, 739–752 (2020).

25. Burke, T., Rowland, C., Whyatt, J. D., Blackburn, G. A. & Abbatt, J. Achieving national scale targets for carbon sequestration through afforestation: Geospatial assessment of feasibility and policy implications. *Environ Sci Policy* **124**, 279–292 (2021).

26. Drever, C. R. *et al.* Natural climate solutions for Canada. *Sci Adv* **7**, eabd6034 (2021).

27. Aguirre-Salado, C. A. *et al.* Improving Identification of Areas for Ecological Restoration for Conservation by Integrating USLE and MCDA in a GIS-Environment: A Pilot Study in a Priority Region Northern Mexico. *Isprs Int Geo-inf* **6**, 262 (2017).

28. Strassburg, B. B. N. *et al.* Global priority areas for ecosystem restoration. *Nature* **586**, 724–729 (2020).

29. Zhang, D. *et al.* A novel similar habitat potential model based on sliding-window technique for vegetation restoration potential mapping. *Land Degrad Dev* **31**, 760–772 (2020).

30. Poccard-Chapuis, R. *et al.* Mapping Land Suitability to Guide Landscape Restoration in the Amazon. *Land* **10**, 368 (2021).

31. Ribeiro, S. M. C. *et al.* A spatially explicit index for mapping Forest Restoration Vocation (FRV) at the landscape scale: Application in the Rio Doce basin, Brazil. *Sci Total Environ* **744**, 140647 (2020).

32. Barnett, A., Fargione, J. & Smith, M. P. Mapping Trade-Offs in Ecosystem Services from Reforestation in the Mississippi Alluvial Valley. *Bioscience* **66**, 223–237 (2016).

33. Chen, M. *et al.* Evaluating suitability of land for forest landscape restoration: A case study of Three Gorges Reservoir, China. *Ecol Indic* **127**, 107765 (2021).

34. Brancalion, P. H. S. *et al.* Global restoration opportunities in tropical rainforest landscapes. *Sci. Adv.* **5**, eaav3223 (2019).

35. Bastin, J.-F. *et al.* The global tree restoration potential. *Science* **365**, 76–79 (2019).

36. Polglase, P. J. *et al.* Potential for forest carbon plantings to offset greenhouse emissions in Australia: economics and constraints to implementation. *Climatic Change* **121**, 161–175 (2013).

37. Greve, M., Reyers, B., Lykke, A. M. & Svenning, J.-C. Spatial optimization of carbon-stocking projects across Africa integrating stocking potential with co-benefits and feasibility. *Nat Commun* **4**, 2975 (2013).

38. Strassburg, B. B. N. *et al.* Strategic approaches to restoring ecosystems can triple conservation gains and halve costs. *Nat Ecol Evol* **3**, 62–70 (2019).

39. Chomphosy, W. H. *et al.* Ecosystem services benefits from the restoration of non-producing US oil and gas lands. *Nat Sustain* **4**, 547–554 (2021).

40. Pittman, K., Hansen, M. C., Becker-Reshef, I., Potapov, P. V. & Justice, C. O. Estimating Global Cropland Extent with Multi-year MODIS Data. *Remote Sens-basel* **2**, 1844–1863 (2010).

41. Dodson, J. E., Bright, E. A., Coleman, P. R., Durfee, R. C. & Worley, B. A. LandScan: A Global Population Database for Estimating Populations at Risk. *Photogrammetric Engineering & Remote Sensing* **7**, 849–857 (2000).

42. Hansen, M. C. *et al.* High-Resolution Global Maps of 21st-Century Forest Cover Change. *Science* **342**, 850–853 (2013).

43. Fritz, S. *et al.* Mapping global cropland and field size. *Global Change Biol* **21**, 1980–1992 (2015).

44. Li, X. *et al.* A New Global Land-Use and Land-Cover Change Product at a 1-km Resolution for 2010 to 2100 Based on Human–Environment Interactions. *Ann. Am. Assoc. Geogr.* **107**, 1040–1059 (2017).

45. Chen, G. *et al.* Global projections of future urban land expansion under shared socioeconomic pathways. *Nat. Commun.* **11**, 537 (2020).

46. Baccini, A. *et al.* Estimated carbon dioxide emissions from tropical deforestation improved by carbon-density maps. *Nat Clim Change* **2**, 182–185 (2012).

47. Ramankutty, N., Evan, A. T., Monfreda, C. & Foley, J. A. Farming the planet: 1. Geographic distribution of global agricultural lands in the year 2000. *Global Biogeochem Cy* **22**, n/a-n/a (2008).

48. Potapov, P. *et al.* Mapping global forest canopy height through integration of GEDI and Landsat data. *Remote Sens Environ* **253**, 112165 (2021).

49. Tubiello, F. N. *et al.* Measuring the world's cropland area. *Nat Food* 1–3 (2023) doi:10.1038/s43016-022-00667-9.

50. Zhang, X. *et al.* GWL_FCS30: a global 30 m wetland map with a fine classification system using multi-sourced and time-series remote sensing imagery in 2020. *Earth Syst Sci Data* **15**, 265–293 (2022).

51. Melton, J. R. *et al.* A map of global peatland extent created using machine learning (Peat-ML). *Geosci Model Dev* **15**, 4709–4738 (2022).

52. Potapov, P. *et al.* The Global 2000-2020 Land Cover and Land Use Change Dataset Derived From the Landsat Archive: First Results. *Frontiers Remote Sens* **3**, 856903 (2022).

53. Shimada, M. *et al.* New global forest/non-forest maps from ALOS PALSAR data (2007–2010). *Remote Sens Environ* **155**, 13–31 (2014).

54. Potapov, P. *et al.* Global maps of cropland extent and change show accelerated cropland expansion in the twenty-first century. *Nat Food* **3**, 19–28 (2022).

55. Pickens, A. H. *et al.* Mapping and sampling to characterize global inland water dynamics from 1999 to 2018 with full Landsat time-series. *Remote Sens. Environ.* **243**, 111792 (2020).

56. Zanaga, D. *et al. ESA WorldCover 10 m 2020 V100*. https://zenodo.org/records/5571936 (2021).

57. Zanaga, D. *et al. ESA WorldCover 10 m 2021 V200*. https://zenodo.org/records/7254221 (2022).

58. Hasler, N. *et al.* Accounting for albedo change to identify climate-positive tree cover restoration. *Nat. Commun.* **15**, 2275 (2024).

59. Cook-Patton, S. C. *et al.* Mapping carbon accumulation potential from global natural forest regrowth. *Nature* **585**, 545–550 (2020).

60. University, C. for I. E. S. I. N.-C.-C. *Gridded Population of World Version 4 (GPWv4), Revision 11*. https://doi.org/10.7927/H4JW8BX5 (2018).

61. Coppedge, M. & Gerring, J. *“V-Dem Global Dataset V13” Varieties of Democracy (V-Dem) Project*. https://doi.org/10.23696/vdemds23 (2023).

62. Dubertret, F. & Wily, L. A. *Percent of Indigenous and Community Lands*. www.landmarkmap.org (2015).

63. Feyertag, J. *et al. Prindex Comparative Report*. https://www.prindex.net/reports/prindex-comparative-report-july-2020/ (2020).

64. Network, C. for I. E. S. I. Global Gridded Relative Deprivation Index (GRDI), Version 1. https://doi.org/10.7927/3xxe-ap97 (2022).

65. Weiss, D. J. *et al.* Global maps of travel time to healthcare facilities. *Nat. Med.* **26**, 1835–1838 (2020).

66. Theobald, D. M., Harrison-Atlas, D., Monahan, W. B. & Albano, C. M. Ecologically-Relevant Maps of Landforms and Physiographic Diversity for Climate Adaptation Planning. *PLoS ONE* **10**, e0143619 (2015).

67. Sewell, A., Löwenhardt, S. van der E., Hannah & Löwenhardt, H. *Goals and Commitments for the Restoration Decade: A Global Overview of Countries’ Restoration Commitments under the Rio Conventions and Other Pledges*. 1–38

https://www.pbl.nl/sites/default/files/downloads/pbl-2020-goals-and-commitments-for-the-restoration-decade-3906.pdf (2020).

68. IUCN, U.-W. and. Protected Planet: The World Database on Protected Areas (WDPA). http://protectedplanet.net/ (2023).

69. Rakotonarivo, O. S. *et al.* Resolving land tenure security is essential to deliver forest restoration. *Commun Earth Environ* **4**, 179 (2023).
